# Link predication based on matrix factorization by fusion of multi class organizations of the network

**DOI:** 10.1038/s41598-017-09081-9

**Published:** 2017-08-21

**Authors:** Pengfei Jiao, Fei Cai, Yiding Feng, Wenjun Wang

**Affiliations:** 1 School of Computer Science and Technology, Tianjin University, Tianjin, 300350 China; 2 School of Surveying and Geo-Informatics, Shandong Jianzhu University, Jinan, 250101 China

## Abstract

Link predication aims at forecasting the latent or unobserved edges in the complex networks and has a wide range of applications in reality. Almost existing methods and models only take advantage of one class organization of the networks, which always lose important information hidden in other organizations of the network. In this paper, we propose a link predication framework which makes the best of the structure of networks in different level of organizations based on nonnegative matrix factorization, which is called *NMF* ^3^ here. We first map the observed network into another space by kernel functions, which could get the different order organizations. Then we combine the adjacency matrix of the network with one of other organizations, which makes us obtain the objective function of our framework for link predication based on the nonnegative matrix factorization. Third, we derive an iterative algorithm to optimize the objective function, which converges to a local optimum, and we propose a fast optimization strategy for large networks. Lastly, we test the proposed framework based on two kernel functions on a series of real world networks under different sizes of training set, and the experimental results show the feasibility, effectiveness, and competitiveness of the proposed framework.

## Introduction

Many real world systems such as social, biological, computer, physical, can be modeled as complex networks^1^. Learning the structure, function and dynamic can help us to understand the formation mechanism, explore the evolution, and forecast the changes of the complex networks^2^. Lots of interesting research hotspots have been proposed, such as community detection^3^, spreading dynamics^4^, cascading reactions^5^, network synchronization^6^ and control^7^. Meanwhile, link predication, has a closeness relation to other research topics and a wide range of applications in reality^8^, which devotes to estimate and predicate the unobserved or latent existent edges between pairs of nodes in the networks based on the observed linked structure. Link predication has been successfully applied to recommendation system^9^, evaluation of network models^10^, analysis of network evolution^11, 12^, the predication of interactions between proteins in biological networks^13^ and so on. The basic and important evidence is that two nodes are more likely linked if they are more similar^14^.

There are a growing number of models and methods for link predication proposing recently^14^. These methods can be divided into three categories in general. The first class and classic methods are similarity-based methods, the hypothesis of which are that nodes are similar only if they are linked similar nodes or close to each other based on the distances denoted on the networks in various ways^8^. Such as the common neighbors (CN) index^15^ and Jaccard index^8^, which are based on the local similarity in the networks, the former denotes the number of common neighbors between the two nodes, the other denotes the ratio of the number of common neighbors and the number of the complete set of neighbors for two nodes. In addition, there are a lot of similarity-based methods, such as global similarity index, Katz^16^, and Quasi-Local index, Local Path Index. The second class methods are probabilistic and statistical approaches, which assume that there are generative mechanisms for the network and these methods build a model to fit the observed structure and estimate model parameters and then compute the linked probability of all the unobserved links in candidate set, such as the hierarchical structure model^17^ and the stochastic block model^18^. The third class are the algorithmic methods, which usually benefit the link predication as a supervised learning or optimization problem. Such as the matrix factorization model^19^, which is usually used in link predication by extract features in the network and is also the foundation of our framework.

However, all most of current link predication methods just take advantage of one class organization of the network. For example, the similarity-based methods just take advantage of one of specific similarity structures, such as common neighbors or Jaccard indexes; the hierarchical structure model, as a classic and popular statistical approach, infers hierarchical structure from the observed network for link predication based on the hierarchical rand graph model; the nonnegative matrix factorization (NMF) method extracts the basis matrix and coefficient matrix based on the observed network, which assumes that each pair nodes are independent, although Krishna *et al*.^19^ considers the similarity-based index as a penalty term adding to the objective function of the NMF, there is no determined interpretations for that. A perturbation-based framework based on NMF^20^ is proposed, which also could join some similarity-based index for link prediction. As we discussed above, whether the similarity-based methods, the probabilistic and statistical approaches, or the algorithmic methods, do not take full advantage of multi class organization of the observed networks in a simple, intuitive, principled, and interpreted way.

How can we construct the different organizations of the complex networks in a principled way? A simple selection is the kernel function^21^, which could enable the data to operate in a high-dimensional, implicit feature space, and it has been successfully applied to neural network and support vector machine. For that in a complex network, we can get various of organization structures of it by mapping the network with different kernel functions, called organization structures, which will help us to explore the structure and promote the performance of link predication in the complex network.

In this paper, we propose a link predication framework which makes the best of the structure of networks in different level of organizations by use of kernel functions. Based on nonnegative matrix factorization, we proposed a framework that combine the adjacency matrix and one class of organization structure in a principled and effective way, which we called *NMF* ^3^ (Nonnegative Matrix Factorization based Fusion Framework). In detail, we first map the observed network into another space by kernel functions. Then we combine the adjacency matrix of the network and one of other organization structures, which makes us obtain the objective function of our framework for link predication based on the nonnegative matrix factorization. Thirdly, we derive an iterative algorithm to optimize the objective function, which converges to a local optimum, and we propose a fast optimization strategy for large networks. Lastly, we test the proposed framework based on two kernel functions on a series of real world networks under different sizes of training set, and the experimental results based on the prediction accuracy show the feasibility, effectiveness, and competitiveness of the proposed framework.

## Results

In this section, we introduce the mathematical definition of link predication, the formation of proposed *NMF* ^3^, evaluation index and experimental results on a series of real world networks.

### Definition of the link predication problem

As most of works about link predication denoted, we consider an unweight and undirected network *G* = (*V*, *E*), *V* and *E* represent the sets of nodes and edges in the network, respectively. *n* = |*V*| and *m* = |*E*| are the number of nodes and edges of this network, respectively. The adjacency matrix of the network is denoted as *A*, if nodes *i* and *j* has a link, then *A*_*ij*_ = *A*_*ji*_ = 1 and *A*_*ij*_ = *A*_*ji*_ = 0, otherwise. As demanded of link predication, we divide the edges of the network into training set and test set, denoted as *E* ^1^ and *E* ^2^, it is obvious that *E* = *E* ^1^ ∪ *E* ^2^ and *E* ^1^ ∩ *E* ^2^ = Ø. We use *A* ^1^ and *A* ^2^ denoting the matrix formation of *E* ^1^ and *E* ^2^ with all the nodes in *V*, respectively, and both of them are symmetric with 1 or 0 as the elements and *A* ^1^ + *A* ^2^ = *A*.

We let *L* = |*E* ^2^|/2 be the number of edges in test set and it is easy to know |*E* ^1^| = 2(*m* − *L*), the number of all the possible edges in the network but out of the training set, we denote it as candidate set, is $|\bar{E}|=n(n-\mathrm{1)/2}-(m-L)$. Then we need to learn one model from the training set *E* ^1^, compute likelihood scores for each edge in the candidate set, select the edges with top *L* values, and validate that on the testing set *E* ^2^ based on some evaluation indexes.

### Formation of proposed *NMF*^3^

Here, we will introduce the formation of our proposed link predication framework, including how to map the network into another space to get the other classes of organization structure of the network based on kernel functions and how to construct our proposed model.

#### Kernel function

Kernel functions have been widely applied to pattern recognition and machine learning, which are based on a fixed nonlinear feature space mapping *ϕ*(*x*), the kernel function is generally given by the relation

1 $$k(x,x^{\prime} )=\varphi {(x)}^{T}\varphi (x^{\prime} )$$

For a given network, we can regard each column of the adjacency matrix, the first order link of one node, as the feature vector of the node. So a series of kernel functions can be applied to the network to get different classes of organization structure. Such as the polynomial kernel, a non-stationary kernel and for problems where all the training data is normalized, the gaussian kernel and exponential kernel, which are examples of radial basis functions, and so on.

Without loss of generality, in this paper, we introduce two classic kernel functions as our instances in the proposed framework, called linear kernel^22^ and covariance kernel^23^. They are denoted on the network as

2 $${K}_{1}(X)={X}^{T}X$$

and

3 $${K}_{2}(X)=\frac{1}{n-1}\sum _{i=1}^{n}({X}_{\cdot i}-\mu ){({X}_{\cdot i}-\mu )}^{T}$$

where, *X* is the adjacency matrix of the observed network (or training set), *X*_·*i*_ is the *i*-*th* column of *X* and *μ* is the average value of all the *X*_·*i*_. It is obvious that both *K*_1_(*X*) and *K*_2_(*X*) are the symmetric positive matrices and there is no additional parameter in both them. The linear kernel *K*_1_(*X*) could extract the local structure informations of the network, yet the covariance kernel *K*_2_(*X*) could extract the global structure information. Although we just take advantage of the two kernel functions in our paper, we believe that the proposed framework can be easily extended and scaled.

#### Detail of the framework

Before introducing our proposed framework, we simply review the nonnegative matrix factorization for link predication. Based on the adjacency matrix *X* of observed network or training set, the objective function can be written as

4 $$\mathop{\min }\limits_{W\ge 0,H\ge 0}O=D(X|WH)+\lambda f(W,H)$$

where *D*(*X*|*WH*) represents the distance between *X* and *WH*, such as the quadratic loss function or *K* − *L* divergence. *W* and *H* are the latent feature matrices(basis matrix and coefficient matrix), with the size of *n* × *C* and *C* × *n*, respectively, *C* is the number of latent features or the inner rank of *X*. *f*(*W*, *H*) is the penalty function about *W* and *H*, such as *L*_1_ or *L*_2_ norm^24^.

Without loss of generality, we consider a simple case with the quadratic loss function, and rewrite the objective function as

5 $$\mathop{\min }\limits_{{W}_{iz}\ge 0,{H}_{zj}\ge 0}O=\sum _{i,j}({X}_{ij}-\sum _{z}({W}_{iz}{H}_{zj}))+\lambda (\sum _{iz}{W}_{iz}^{2}+\sum _{zj}{H}_{zj}^{2})$$

or in a matrix form as

$$\mathop{\min }\limits_{W\ge 0,H\ge 0}O={|X-WH|}_{F}^{2}+\lambda ({|W|}_{F}^{2}+{|H|}_{F}^{2})$$

How to fuse the other organization structure with equation 5 in a principled way? Motivated by the nonnegative matrix factorization for recommendation system, we propose the objective function of the *NMF* ^3^ as follows

6 $$\mathop{\min }\limits_{{W}_{iz}\ge 0,{H}_{zj}\ge 0}O=\sum _{i,j}(1+\gamma {R}_{ij})({X}_{ij}-\sum _{z}({W}_{iz}{H}_{zj}))+\lambda (\sum _{iz}{W}_{iz}^{2}+\sum _{zj}{H}_{zj}^{2})$$

where *R* is the organization structure obtained by the kernel function, which has the same size with *X*. Parameter *γ* is used to scale the strength of *R*. After optimizing the equation 6, we can compute the similarity of all the edges in candidate set by *WH*. The setting of parameters *γ* and *λ* is in the experimental results, and the how to optimize the objective function, the detail algorithm and how to scale it to large networks can be seen in section *methods*.

### Evaluation index

To quantify the performance of the link predication methods, we introduce three evaluation metric, area under the receiver operating characteristic curve (AUC)^25^, Precision^26^ and Prediction-Power^27^. In fact, link predication methods give an order list of all the edges in candidate set $\bar{E}$ according the computing similarity values.

Based on the rank of edges in $\bar{E}$, the AUC value is the probability that we randomly select an edge in test set *E* ^2^ with a higher rank score than a randomly selecting an edge in candidate set $\bar{E}$. The AUC can always be approximately calculated as

7 $$AUC=\frac{t^{\prime} +0.5t^{\prime\prime} }{t}$$

where *t*, *t*′ and *t*′′ are the number of times of randomly independent comparisons, a higher score of an edge in test set *E* ^2^ and both two having a same score.

If we select the edges with the top *L* similarity values, denoted as *E* ^*p*^, then the Precision can be computed as

8 $$Precision=\frac{|{E}^{2}\cap {E}^{p}|}{L}$$

which represents the accuracy of link predication methods.

As discussed in ref. 27, the Prediction-Power (PP) is denoted as

9 $$PP={\mathrm{log}}_{10}\frac{Precision}{Precisio{n}_{Random}}$$

where *Precision*_*Random*_ is the performance of random-predictor can be computed by *L*/(*n*(*n* − 1)/2 − (*m* − *L*)), and this metric can assess deviation from the mean random-predictor performance.

### Baseline methods

We compare our method with several well known methods, including CN, AA, RA, Salton, Jaccard, ACT and CRA indices and some widely used global methods, which are denoted as following, respectively.

1. Common Neighbors (*CN*)^15^, which is denoted between nodes *x* and *y* as
  10 $${s}_{xy}^{CN}=|{\rm{\Gamma }}(x)\cap {\rm{\Gamma }}(y)|$$
  where Γ(*x*) denotes the set of neighbors of node *x*.
2. Adamic-Adar (*AA*)^28^, which is denoted as
  11 $${s}_{xy}^{AA}=\sum _{z\in {\rm{\Gamma }}(x)\cap {\rm{\Gamma }}(y)}\frac{1}{\mathrm{log}\,{k}_{z}}$$
  where *k*_*z*_ is the degree of node *z*. This index considers the information about the degree of the common neighbors of the two nodes, and assigns the less-connected neighbors more weight.
3. Resource Allocation (*RA*)^29^, which is denoted as
  12 $${s}_{xy}^{RA}=\sum _{z\in {\rm{\Gamma }}(x)\cap {\rm{\Gamma }}(y)}\frac{1}{{k}_{z}}$$
  the RA index assigns the different weight to the common neighbors.
4. *Salton* Index^30^, which is denoted as
  13 $${s}_{xy}^{Salton}=\frac{|{\rm{\Gamma }}(x)\cap {\rm{\Gamma }}(y)|}{\sqrt{{k}_{x}{k}_{y}}}$$
  the index is also based on the number of common neighbors yet with another normalization methods.
5. *Jaccard* index^8^, which is denoted as
  14 $${s}_{xy}^{Jaccard}=\frac{|{\rm{\Gamma }}(x)\cap {\rm{\Gamma }}(y)|}{|{\rm{\Gamma }}(x)\cup {\rm{\Gamma }}(y)|}$$
  which is the ratio between the number of the intersection of Γ(*x*) and Γ(*y*) and the number of the union of that.
6. Average Commute Time (*ACT*)^31^, it is denoted as
  15 $${s}_{xy}^{ACT}=\frac{1}{{l}_{xx}^{+}+{l}_{yy}^{+}-2{l}_{xy}^{+}}$$
  which means that two nodes are more similar if they have a smaller average commute time, and this similarity between the nodes *x* and *y* can be defined as the reciprocal of average commute time between *x* and *y*. Where ${l}_{xx}^{+}$ represents the elements of matrix *L* ^+^, the pseudo inverse of the Laplacian matrix of the network.
7. CRA, which is a extend similar index based on the RA denoted by Carlo Vittorio Cannistraci in ref. 27. It is denoted as
  16 $${s}_{xy}^{CRA}=\sum _{z\in {\rm{\Gamma }}(x)\cap {\rm{\Gamma }}(y)}\frac{{\alpha }_{z}}{{k}_{z}}$$
  where *α*_*z*_ refers to the sub-set of neighbors of *z* that are also common neighbors of nodes *x* and *y*.
8. SPM, which is called structural perturbation method^32^ for link predication by assuming that the regularity of a network is reflected in the consistency of structural features before and after a random removal of a small set of links.
9. HSM, the hierarchical structure model proposed by Aaron Clauset in ref. 17, which can infer hierarchical structure from network data and predict the missing links.
10. SBM, which can deal the data reliability in complex networks and infer missing and spurious links based on the stochastic block model^18^.
11. LR, a robust principal component analysis based^33^ for estimate the missing links in complex networks and we set the weighting parameter to balance the low-rank property and sparsity as 0.1.
12. LOOP, which is an algorithmic framework of probability by denoting a predefined structural Hamiltonian^34^ based on the network organizing, and predict each non-observed link by computing the conditional probability of adding the link to the observed network. As far as we know, the methods LOOP and SPM have nearly best performance on link predication recently, however, both of two methods are time-consuming, especially the LOOP.

In this paper, we denote *NMF* ^3^ − 1 and *NMF* ^3^ − 2 indicating the linear kernel and and covariance kernel based on the proposed framework.

## Experimental results

### Datasets

We evaluate the performance of our proposed framework by using ten quality networks from various areas, including social, biological, and technological network. The networks used in the experiment are described as follows and the basic statistical features are shown in Table 1. Directed links are treated as undirected, multiple links are treated as a single unweighted link and self loops are removed.

1. Jazz^35^: A collaboration network of jazz musicians consists of 198 nodes and 2742 interactions.
2. USAir^32^: The air transportation network of USA consists of 332 nodes and 2126 links. The nodes of the network are airports, and each edge represents one airline.
3. NetScience^36^: A coauthor-ship network of scientists working on network theory and experiment consists of 379 nodes and 914 links. The nodes of the network are the scientists, and each edge represents the cooperative relationship between the scientists.
4. Politicablogs^37^: The network of American political blogosphere consists of 1222 nodes and 19021 links. The nodes of the network are blog page, and each edge represents the hyperlinks between the blog pages.
5. Router^38^: A snapshot of the structure of the Internet at the level of autonomous systems consists of 5022 nodes and 6258 links.
6. Celegans^39^: Neural network of elegans consists of 297 nodes and 2148 links. The nodes of the network are neurons, and each edge represents the gap junction between neurons.
7. Yeas^40^: A protein-protein interaction network in budding yeast consists of 2375 nodes and 11693 interactions. The node of the network is protein, and the link represents its interactions.
8. Metabolic^41^: A metabolic network of C. elegans consists of 453 nodes and 2025 interactions.
9. FWFD http://vlado.fmf.uni-lj.si/pub/networks/data/bio/foodweb/foodweb.html: A food web in Florida Bay during the day season. The network contains 128 species of dry season and 2137 interactions.
10. FWMW http://vlado.fmf.uni-lj.si/pub/networks/data/bio/foodweb/foodweb.html: A food web in Mangrove Estuary during the wet season consists of 97 nodes and 1493 interactions.

**Table 1:** The basic topological features of ten real networks studied in this paper, where |*V*| and |*E*| are the numbers of nodes and links, 〈*k*〉 is the average degree, *CC* is the clustering coefficient and 〈*d*〉 is the average shortest distance. *H* is the degree heterogeneity, as $H=\frac{\langle {k}^{2}\rangle }{{\langle k\rangle }^{2}}$, and *r* is the assortative coefficient.

Networks	|*V*|	|*E*|	〈*k*〉	*CC*	〈*d*〉	*H*	*r*
Jazz	198	2742	27.700	0.618	2.235	1.395	0.020
USAir	332	2126	12.810	0.749	2.740	3.460	0.208
NetSci	379	914	4.820	0.798	6.040	1.660	0.082
PB	1222	16714	27.360	0.360	2.740	2.970	0.221
Router	5022	6258	2.490	0.033	6.450	5.500	0.138
C. elegans	297	2148	14.470	0.308	2.460	1.800	0.163
Yeast	2375	11693	9.850	0.388	5.100	3.480	0.454
Metabolic	453	2025	8.940	0.647	2.664	4.485	0.226
FWFD	128	2075	32.442	0.335	1.776	1.237	0.112
FWMW	97	1446	29.814	0.468	1.693	1.266	0.151

### Results and analysis

**Figure 1:**
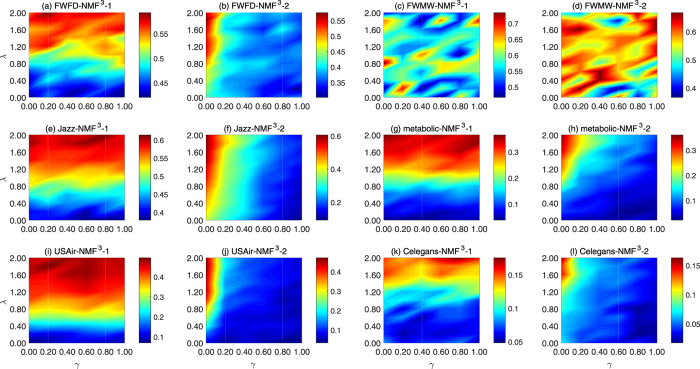
Parameter sensitivity: we conducted the experiments of parameter sensitivity on six networks. We vary the number of *γ* and the *λ* to determine their impact on the network link prediction. The Each data point is averaged over 100 independent runs.

Parameters setting: we select six networks including FWFD, FWMW, Jazz, Metabolic, USAir and Celegans from the all ten networks, and analyze the experimental sensitivity of *γ* and *λ* in our framework with the performance of link predication. As represented in Fig. 1, we set the proportion of training set as 0.9, and take the widely used evaluation index *Precision* for link predication as evidence. It is obvious that the performances on FWFD, Jazz, Metabolic, USAir and Celegans are gradual stable. Although the different settings of *γ* and *λ* have significant influence on the predict results, we also know that our framework has equally better performance than other baseline methods. Without losing generality, we set *γ* = 0.1 and *λ* = 2 in subsequent experiments.

As represented in Tables 2, 3 and 4, we show the performance on the ten real world networks with the proportion of training set 0.9 based on *AUC*, *Precision* and *PP*, respectively. The black texts represent the largest value in each column, each row in the table represents the experimental results of one method including the average value and standard deviation over 100 times of dividing the network into training set and test set. From Table 2, our method *NMF* ^3^, SPM and LOOP have more competitive performance based on precision compared with other methods. As represented in Table 3, our method and SPM have better performance based on AUC, and the RA index is a best select in similarity-based index. In Table 4, we also show the mean value of *PP* of each method at last column. Also of note, the *N*/*A* in the tables represents that the value could not be computed for the corresponding method not applicable to large-scale networks. In general, our methods have nearly equal performance to LOOP and SPM based on the three evaluation index, however, the both two methods are more time-consuming compared with the proposed method in this paper and we analyze the computational complexity in section *methods*.

**Table 2:** Link prediction accuracy is measured by precision on the 10 real networks. We compared our methods (*NMF* ^3^ − 1, *NMF* ^3^ − 2) with other methods on the 10 network data sets and the precision is returned with an average run of over 100 times. For each data set, the presented links are partitioned into training set (90%) and test set (10%).

Precision(0.9)	Celegans	FWMW	FWFD	Jazz	metabolic	USAir	NetScience	Politicalblogs	Router	Yeast
*NMF* ^3^ − 1	0.152	0.660	**0.581**	0.620	0.343	**0.469**	0.327	0.171	**0.174**	0.537
	0.022	0.114	0.021	0.008	0.021	0.013	0.024	0.007	0.008	0.015
*NMF* ^3^ − 2	0.131	**0.700**	0.530	0.560	0.316	0.398	0.338	0.119	0.160	0.408
	0.025	0.122	0.026	0.019	0.023	0.010	0.019	0.009	0.024	0.014
CRA	0.144	0.033	0.083	0.559	0.204	0.406	0.321	0.179	0.033	0.119
	0.008	0.058	0.018	0.023	0.021	0.023	0.039	0.007	0.013	0.015
CN	0.108	0.000	0.076	0.508	0.133	0.383	0.330	0.171	0.024	0.121
	0.011	0.000	0.005	0.046	0.021	0.042	0.046	0.005	0.000	0.018
AA	0.125	0.000	0.081	0.532	0.194	0.415	0.542	0.168	0.026	0.104
	0.014	0.000	0.005	0.035	0.008	0.036	0.021	0.004	0.003	0.015
RA	0.104	0.000	0.082	0.543	0.281	**0.469**	**0.736**	0.145	0.011	0.115
	0.019	0.000	0.005	0.028	0.023	0.033	0.013	0.004	0.000	0.006
Salton	0.042	0.000	0.009	0.537	0.049	0.059	0.320	0.007	0.036	0.063
	0.005	0.000	0.013	0.047	0.013	0.016	0.007	0.005	0.003	0.001
Jaccard	0.063	0.000	0.008	0.522	0.049	0.078	0.301	0.016	0.018	0.031
	0.003	0.000	0.014	0.046	0.013	0.015	0.015	0.003	0.001	0.003
ACT	0.063	0.000	0.153	0.167	0.082	0.329	0.190	0.070	0.026	0.124
	0.015	0.000	0.005	0.056	0.023	0.019	0.012	0.004	0.009	0.016
SPM	0.133	0.545	0.570	0.667	0.315	0.454	0.596	**0.233**	0.004	**0.788**
	0.025	0.122	0.026	0.019	0.023	0.010	0.019	0.009	0.024	0.014
HSM	0.085	0.440	0.261	0.325	0.109	0.142	0.299	0.107	0.064	0.081
	0.005	0.002	0.002	0.026	0.019	0.011	0.015	0.003	0.002	0.012
SBM	0.145	0.601	0.417	0.410	0.197	0.335	0.178	0.110	0.156	0.122
	0.004	0.001	0.003	0.031	0.015	0.012	0.009	0.004	0.003	0.015
LR	0.138	0.050	0.537	0.559	0.208	0.399	0.069	0.074	0.054	0.468
	0.006	0.002	0.002	0.026	0.019	0.011	0.015	0.003	0.002	0.012
LOOP	**0.181**	0.200	0.564	**0.685**	**0.394**	0.466	N/A	N/A	N/A	N/A
	0.003	0.001	0.002	0.030	0.014	0.015	N/A	N/A	N/A	N/A

**Table 3:** Link prediction accuracy measured by AUC on the 10 real networks. We compared our methods (*NMF* ^3^ − 1, *NMF* ^3^ − 2) with other methods on the 10 network data sets and the AUC are returned with an average run of over 100 times. For each data set, the presented links are partitioned into training set (90%) and test set (10%).

AUC(0.9)	Celegans	FWMW	FWFD	Jazz	metabolic	USAir	NetScience	Politicalblogs	Router	Yeast
*NMF* ^3^ − 1	**0.908**	**0.996**	0.956	0.960	0.918	0.956	0.791	0.951	0.703	**0.972**
	0.024	0.005	0.017	0.014	0.035	0.032	0.032	0.015	0.017	0.009
*NMF* ^3^ − 2	0.894	0.984	**0.960**	0.956	0.910	0.944	0.821	0.938	0.751	0.969
	0.021	0.036	0.014	0.030	0.022	0.023	0.024	0.018	0.021	0.012
CRA	0.782	0.500	0.645	0.982	0.867	0.935	0.827	0.900	0.533	0.872
	0.052	0.000	0.061	0.003	0.019	0.020	0.008	0.018	0.013	0.019
CN	0.823	0.375	0.582	0.940	0.920	0.960	0.983	0.932	0.527	0.880
	0.016	0.009	0.058	0.009	0.023	0.015	0.008	0.015	0.009	0.012
AA	0.890	0.370	0.607	0.967	**0.967**	0.965	0.988	0.910	0.534	0.879
	0.035	0.013	0.067	0.015	0.020	0.013	0.003	0.046	0.015	0.018
RA	0.872	0.390	0.583	**0.990**	0.952	0.975	0.993	0.918	0.529	0.884
	0.008	0.010	0.029	0.010	0.008	0.025	0.006	0.021	0.013	0.016
Salton	0.802	0.383	0.547	**0.990**	0.805	0.922	0.995	0.887	0.540	0.870
	0.032	0.032	0.104	0.000	0.026	0.037	0.005	0.040	0.017	0.030
Jaccard	0.793	0.400	0.510	0.970	0.770	0.882	0.995	0.872	0.526	0.885
	0.008	0.018	0.115	0.030	0.031	0.032	0.005	0.028	0.010	0.022
ACT	0.750	0.483	0.700	0.787	0.757	0.900	0.613	0.903	**0.918**	0.910
	0.046	0.060	0.044	0.012	0.021	0.040	0.051	0.012	0.034	0.023
SPM	0.833	**0.996**	0.867	0.967	**0.967**	0.967	**0.998**	**0.997**	0.585	0.930
	0.016	0.009	0.058	0.009	0.023	0.015	0.008	0.015	0.009	0.012
HSM	0.850	0.940	0.821	0.912	0.815	0.855	0.810	0.851	0.709	0.674
	0.025	0.010	0.025	0.056	0.023	0.019	0.012	0.024	0.019	0.016
SBM	0.860	0.984	0.941	0.933	0.908	0.945	0.899	0.891	0.910	0.770
	0.031	0.009	0.035	0.152	0.030	0.021	0.018	0.018	0.021	0.023
LR	0.573	0.550	0.906	0.886	0.585	0.800	0.570	0.515	0.535	0.800
	0.006	0.002	0.002	0.026	0.019	0.011	0.015	0.003	0.002	0.012
LOOP	0.901	0.815	0.955	0.978	0.965	**0.976**	N/A	N/A	N/A	N/A
	0.004	0.001	0.003	0.031	0.015	0.012	N/A	N/A	N/A	N/A

**Table 4:** Link prediction accuracy measured by Prediction-Power on the 10 real networks. We compared our methods (*NMF* ^3^ − 1, *NMF* ^3^ − 2) with other methods on the 10 network data sets and the AUC are returned with an average run of over 100 times.

PP	Celegans	FWMW	FWFD	Jazz	metabolic	USAir	NetScience	Politicalblogs	Router	Yeast	*mean*
*NMF* ^3^ − 1	1.472	1.184	1.235	1.585	2.230	2.067	2.401	1.874	3.544	3.110	2.070
*NMF* ^3^ − 2	1.409	1.209	1.195	1.540	2.195	1.996	2.415	1.716	3.508	2.991	2.017
CRA	1.357	−0.117	0.417	1.553	2.041	1.985	2.393	1.894	2.822	2.456	1.680
CN	1.172	−1.636	0.350	1.498	1.819	1.980	2.405	1.874	2.684	2.463	1.461
AA	1.267	−1.636	0.377	1.518	1.982	2.014	2.620	1.866	2.719	2.397	1.512
RA	1.320	−1.636	0.385	1.527	2.143	2.068	2.753	1.802	2.345	2.441	1.515
Salton	0.737	−1.636	−0.553	1.522	1.387	1.170	2.391	0.486	2.860	2.180	1.055
Jaccard	0.760	−1.636	−0.632	1.510	1.387	1.290	2.365	0.845	2.559	1.872	1.032
ACT	1.061	−1.636	0.656	1.015	1.609	1.913	2.165	1.486	2.719	2.474	1.346
SPM	1.416	1.101	1.207	1.616	2.194	2.053	2.662	2.008	1.906	3.277	1.944
HSM	1.220	1.008	0.887	1.304	1.732	1.548	2.362	1.670	3.110	2.289	1.713
SBM	1.452	1.143	1.091	1.405	1.724	1.921	2.137	1.682	3.497	2.467	1.852
LR	1.431	0.063	1.200	1.539	2.013	1.997	1.725	1.510	3.036	3.051	1.757
LOOP	1.549	0.665	1.222	1.628	2.290	2.065	N/A	N/A	N/A	N/A	1.570

For each data set, the presented links are partitioned into training set (90%) and test set (10%).

**Figure 2:**
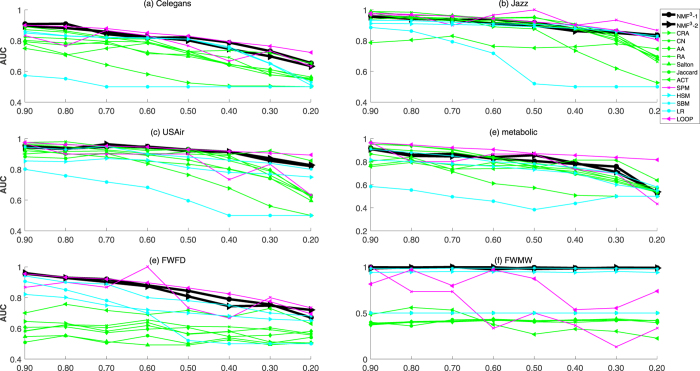
The comparison of AUC of six networks under different fractions of missing links. Besides our kernel framework (*NMF* ^3^ − 1, *NMF* ^3^ − 2), we further compare our methods with eight well-known methods (AA, RA, CN, Salton, ACT, Jaccard, CRA, SPM, HSM, SBM, LP, LOOP). Each data point is averaged over 100 independent runs.

**Figure 3:**
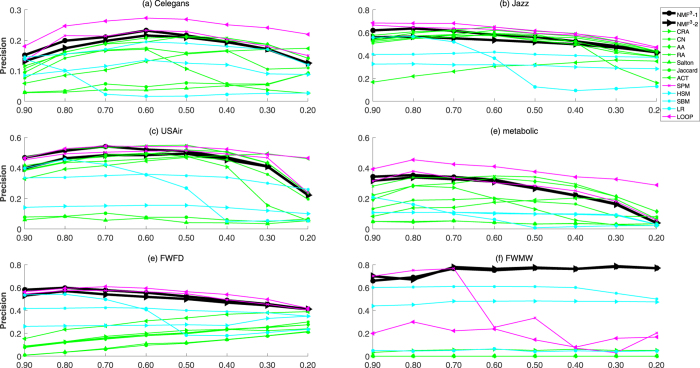
The comparison of precision of six networks under different fractions of missing links. Besides our kernel framework (*NMF* ^3^ − 1, *NMF* ^3^ − 2), we further compare our methods with eight well-known methods (AA, RA, CN, Salton, ACT, Jaccard, CRA, SPM, HSM, SBM, LP, LOOP). Each data point is averaged over 100 independent runs.

**Figure 4:**
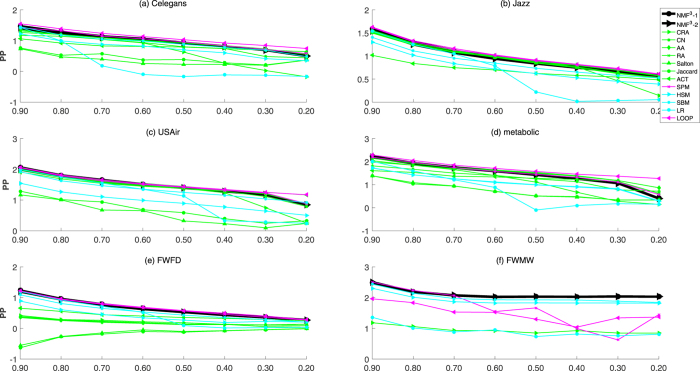
Legend (The comparison of Prediction-Power of six networks under different fractions of missing links. Besides our kernel framework (*NMF* ^3^ − 1, *NMF* ^3^ − 2), we further compare our methods with eight well-known methods (AA, RA, CN, Salton, ACT, Jaccard, CRA, SPM, HSM, SBM, LP, LOOP). Each data point is averaged over 100 independent runs.

Furthermore, we analyze the experimental results on the networks with different fraction of training set from 0.9 to 0.2. As reported in Figs 2, 3 and 4, we show the results of Celegans, Jazz, USAir, Metabolic, FWFD and FWMW based on *AUC*, *Precision* and *PP*, respectively (For that it is time-consuming for global methods for larger networks, especially for the SPM and LOOP). The black lines represent the performance of the proposed *NMF* ^3^ − 1 and *NMF* ^3^ − 2 methods, the purple lines correspond to the SPM and LOOP methods, the rest lines are the other global methods (HSM, SBM, and LR) and similarity-based methods. From the results, it is obvious that SPM, LOOP and our methods have better and competitive performance that others. There are two different expressions in the figures, they are the FWFD and FWMW networks, on which our proposed framework has super performance than other methods. In fact, our methods have competitive and stable performance.

## Discussion

In this paper, we have proposed a framework of link predication which could make multi class organizations of the network. We take two kernel functions as the special cases of the proposed framework and experiments show the feasibility, effectiveness, and competitiveness of the framework.

As an extension to the nonnegative matrix factorization, our proposed framework for link predication not only inherits the advantages of which, but also take full advantage of multi organizations of the network based on kernel function. Furthermore, we proposed a gradient descent algorithm to optimize the object function and extend it to large networks. Other more, our framework is easy to be extended to directed and weighted networks, for that it is based on nonnegative matrix factorization, just by letting the *X* be directed and weighted. And we believe that this proposed method highlights the research in which taking different structure information for link predication.

There are some limitations and improved studies for our proposed framework in future. One of which is how to set parameters *γ* and *λ* to be adaptive on different networks. For our framework only taking the adjacency matrix and one of other organization of the network, making the best of more classes of structure information of the network in a principled and effective way is our next work.

## Methods

In this section, we introduce how to optimize the objective function 6 with a gradient descent algorithm, give a simple operation process for the algorithm and propose a strategy to scale the algorithm for larger networks.

### Parameter learning

The determination of the number of latent features *C* is a very important and difficult problem in the matrix factorization. Here, for it is not our primary attention, we take an easy and effective method for automatic determination of *C*, *Colibri* ^42^, which seeks a nonorthogonal basis by sampling the columns of the input matrix.

Because of the non convex of objective function 6, we alternate update *W* with fixed *H* and update *H* with fixed *W* under the Majorization-Minimization framework^43^. We rewrite the objective function 6 as

17 $$\mathop{\min }\limits_{W\ge 0,H\ge 0}O={|(1+\gamma R)\cdot (X-WH)|}_{F}^{2}+\lambda ({|W|}_{F}^{2}+{|H|}_{F}^{2})$$

Here, the ⋅ represents the element wise multiplication. To enforce the non-negativity constraints of *W* and *H*, we introduce the Lagrangian and write the equation 6 as

18 $$O={|\mathrm{(1}+\gamma R)\cdot (X-WH)|}_{F}^{2}+\lambda ({|W|}_{F}^{2}+{|H|}_{F}^{2})+Tr({\rm{\Phi }}{W}^{T})+Tr({\rm{\Psi }}H)$$

where Φ and Ψ are the Lagrange multipliers, following the Karush- Kuhn-Tucker (KKT) optimality conditions^44^, we set $\frac{\partial O}{\partial W}=\frac{\partial O}{\partial H}=0$, and get

19 $${\rm{\Phi }}=(1+\gamma R)\cdot (-2X{H}^{T}+2WH{H}^{T})+2W$$

and

20 $${\rm{\Psi }}=(1+\gamma R)\cdot (-2{X}^{T}W+2{H}^{T}{W}^{T}W)+2{H}^{T}$$

Then the KKT complimentary slackness conditions yield

21 $${((1+\gamma R)\cdot (-2X{H}^{T}+2WH{H}^{T})+2W)}_{iz}{W}_{iz}=0$$

and

22 $${\mathrm{((1}+\gamma R)\cdot (-2{X}^{T}W+2{H}^{T}{W}^{T}W)+2{H}^{T})}_{jz}{H}_{jz}^{T}=0$$

Following the works^45, 46^, we can easy get the update rules of *W* and *H* as

23 $${W}_{iz}\leftarrow {W}_{iz}\frac{{\mathrm{((1}+\gamma R)\cdot X{H}^{T})}_{iz}}{{\mathrm{((1}+\gamma R)\cdot (WH{H}^{T})+W)}_{iz}}$$

and

24 $${H}_{jz}^{T}\leftarrow {H}_{jz}^{T}\frac{{((1+\gamma R)\cdot {X}^{T}W)}_{jz}}{{((1+\gamma R)\cdot {H}^{T}{W}^{T}W+2{H}^{T})}_{jz}}$$

which makes the objective function 6 converge to a local minimum.

### Algorithm for *NMF*^3^

Here, we summed the algorithm for proposed *NMF* ^3^ based on the procedure of link predication in 1.

**Algorithm 1:**
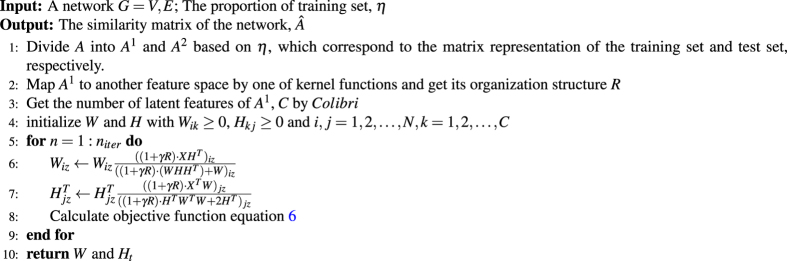
Algorithm for the *NMF* ^3^ framework based on the procedure of link predication.

### Complexity analysis and discussion

Here, we give a simple complexity analysis of the proposed algorithm. The most time-consuming parts are updating *W* and *H*, for each iteration, the time cost of (*γR* · *X*)*H* ^*T*^ is *O*(*N* ^2^ *C* + *N* ^2^), the time cost of ((1 + *γR*) · (*WHH* ^*T*^) + *W*) is *NC* ^2^ + *N* ^2^, so the total time cost of of the algorithm is *O*(*N*_*iter*_(*N* ^2^ *C* + *NC* ^2^ + *N* ^2^ + *NC*)) ~ *O*(*n*_*iter*_(*N* ^2^ *C*)), where *n*_*iter*_ is the number of iterations. If we consider the sparse of real world networks, the time cost can be as *O*(*n*_*iter*_(*mC*)), where *m* is the number of the edge of the network. The most confused problem of the algorithm is that it just converges to a local minimum, so we need run the algorithm many times and chose a best one which has the least value of the objective function.

### Scale to large networks

In order to deal the large networks, we rewritten the object function 6 as

25 $$\mathop{\min }\limits_{{W}_{iz}\ge 0,{H}_{zj}\ge 0}O=\sum _{i\sim j}(1+\gamma {R}_{ij})({X}_{ij}-\sum _{z}({W}_{iz}{H}_{zj}))+\lambda (\sum _{iz}{W}_{iz}^{2}+\sum _{zj}{H}_{zj}^{2})$$

here, the *i* ~ *j* indicates there existence an edge between nodes *i* and *j*, then we could only compute the observed links in the training set of the network. The optimization process of function 25 is similar to the algorithm 1.
